# Co-Amorphous Ursolic Acid–Quercetin Complex Improves Solubility and In Vivo Expectorant–Antitussive Activity

**DOI:** 10.3390/plants15162443

**Published:** 2026-08-11

**Authors:** Yuhang Jiang, Zehong Qiu, Biaoshen Lin, Zhongping Yang, Yanping Hong

**Affiliations:** 1 College of Life Science, Longyan University, Longyan 364000, China; 2 Fujian Provincial Key Laboratory of Preventive Veterinary Medicine and Biotechnology, Longyan University, Longyan 364000, China

**Keywords:** co-amorphous system, ursolic acid, quercetin, loquat leaf, water solubility, cough-relieving and phlegm-resolving

## Abstract

This study investigated the formation of a co-amorphous complex between ursolic acid and quercetin as a chemically defined model system for improving the formulation performance of a poorly soluble triterpenoid acid. Co-amorphous systems were prepared by solvent evaporation and physical grinding at different mass ratios. The optimal formulations were identified and evaluated by water-solubility measurement, powder X-ray diffraction (PXRD), Fourier-transform infrared spectroscopy (FTIR), and in vivo expectorant–antitussive assays. The solvent-derived system showed higher water solubility than the ground system. PXRD and FTIR results suggested that the solvent method more effectively promoted loss of crystallinity and probable intermolecular interactions, whereas the grinding method largely retained the crystalline features of ursolic acid. Both ursolic acid–quercetin formulations exhibited enhanced expectorant and antitussive activities compared with pure ursolic acid. Although quercetin may contribute intrinsic pharmacological activity, the overall improvements were broadly consistent with the altered physicochemical properties of the co-amorphous systems, which may facilitate ursolic acid delivery. As a secondary exploratory comparison, plant-derived triterpenoid-acid-rich and flavonoid-rich fractions from *Eriobotrya japonica* leaves were also co-processed; however, because these fractions were not compositionally characterized, their results were interpreted cautiously and were not used as the primary basis for mechanistic conclusions. Overall, the present study demonstrates that co-amorphous complexation of ursolic acid and quercetin is a promising strategy for improving the solubility-related properties and in vivo activity of a poorly soluble triterpenoid acid.

## 1. Introduction

The dried leaves of *Eriobotrya japonica* (Thunb.) Lindl., a plant belonging to the genus Eriobotrya of the Rosaceae family, are a traditional Chinese herbal medicine. Clinically, they are used to treat symptoms such as lung heat cough, shortness of breath with upward qi reversal, stomach heat with vomiting and upward qi reversal, restlessness, heatiness, and thirst (National Pharmacopoeia Commission 2020). Among its components, triterpenoid acids are effective ingredients that align with its functions and therapeutic uses [1]. They can prolong the cough latency period and have a certain expectorant effect [2], while also demonstrating good safety [3]. However, the main triterpenoid acid active ingredients in the leaves of *E. japonica*, ursolic acid and oleanolic acid, both belong to drugs with low solubility and low permeability. In the Biopharmaceutics Classification System (BCS), they are classified as Class IV drugs, and their poor water solubility seriously hinders the exertion of their biological activity.

In pharmaceutical research, converting poorly soluble substances from a crystalline state to an amorphous state can enhance their solubility in water. Coamorphous systems (CAM) are a potential drug delivery system [4,5] that can improve drug solubility [6,7,8], and they are a highly promising new formulation technology [9]. Particularly in the form of drug–drug CAM, they can be used as combination therapy drugs. They not only increase the water solubility and dissolution rate of two poorly soluble drugs but also enhance efficacy and reduce drug toxic side effects. Some systems even have the potential for synergistic drug release. Ursolic acid is a major pentacyclic triterpenoid acid in *E. japonica* leaves [10] and exhibits various biological activities, including anticancer, antiviral, anti-inflammatory, antioxidant, antifungal, antibacterial, and antidiabetic effects [11]. However, due to its extremely low solubility in water, its bioavailability is affected [12]. Quercetin is a major flavonoid compound in loquat leaves [13], possessing anti-inflammatory, analgesic, and antitussive activities [14]. Additionally, owing to its antibacterial, antiviral, anti-inflammatory, antioxidant properties and strong immune regulatory effects, it may have therapeutic potential against SARS-CoV-2 [15]. However, quercetin and other flavonoids are also extremely difficult to dissolve in water. Literature reports indicate that flavonoids such as quercetin have the ability to form glassy states; the ketones and hydroxyl groups around the flavanol skeleton can provide possibilities for intermolecular interactions, enabling effective combination with drugs to form CAM [5].

In the present study, ursolic acid and quercetin were selected as representative triterpenoid and flavonoid model compounds associated with *E. japonica* leaves, and their co-amorphous system was investigated as the primary focus of this work. The ursolic acid–quercetin formulations were characterized by FTIR and PXRD, and their water solubility together with in vivo expectorant and antitussive activities was evaluated to determine whether co-amorphous complexation could improve the formulation performance of a poorly soluble triterpenoid acid. In addition, plant-derived triterpenoid-acid-rich and flavonoid-rich fractions from *E. japonica* were included only as a preliminary exploratory comparison. Because these fractions were not compositionally characterized in detail, they are not interpreted in this study as chemically equivalent to the ursolic acid–quercetin model system.

## 2. Materials and Methods

### 2.1. Preparation of Amorphous Complex of Ursolic Acid and Quercetin

Co-amorphous complexes were prepared using both physical grinding and solvent methods. In the physical grinding method, ursolic acid (product number U118635, CAS No. 77-52-1, purity ≥ 98%, supplied by Aladdin Chemical Reagent Corporation, Shanghai, China) and quercetin (product number Q111273, CAS No. 117-39-5, purity ≥ 98%, supplied by Aladdin Chemical Reagent Corporation, Shanghai, China) were mixed according to mass ratios of 10:1, 10:2, 10:3, 10:4, and 10:5. Subsequently, a small volume of anhydrous ethanol was added, and the mixture was wet ground using a planetary ball mill (QM-3SP2, Nanjing Tanshin Instrument Equipment Co., Ltd., Nanjing, China) for 2 h, yielding the solid complex. For the solvent method, ursolic acid and quercetin were precisely weighed in mass ratios of 50:1, 40:1, 20:1, 10:1, and 5:1. Ursolic acid was dissolved in anhydrous ethanol under heating at 90 °C using a constant-temperature heating magnetic stirrer (DF-101S, Gongyi Yingyu Instrument Factory, Gongyi, China). Quercetin was separately dissolved in anhydrous ethanol using a 50 °C water bath (DK-98-IIA, Tianjin Taist Instrument Co., Ltd., Tianjin, China). The two solutions were then combined and stirred for 10 min using the same magnetic stirrer. The resulting mixture was concentrated under reduced pressure using a rotary evaporator (RE-52AA, Shanghai Yarong Biochemical Instrument Co., Ltd., Shanghai, China), producing the solid co-amorphous complex.

### 2.2. Exploratory Preparation of Co-Processed Systems from the Triterpenoid-Acid-Rich Fraction and Flavonoid-Rich Fraction of E. japonica Leaves

Dried leaves of *E. japonica* were powdered and extracted with aqueous ethanol under heating. After filtration, the combined extracts were concentrated under reduced pressure using a rotary evaporator (RE-52AA, Shanghai Yarong Biochemical Instrument Co., Ltd., Shanghai, China) to obtain the crude ethanol extract. The crude extract was then suspended in distilled water and fractionated according to polarity to enrich the triterpenoid-acid and flavonoid fractions, following previously reported methods with appropriate modifications [16,17]. Briefly, the concen Ctrated ethanol extract was dispersed in water and successively partitioned with petroleum ether and ethyl acetate. The ethyl acetate-soluble fraction, which is known to be enriched in flavonoids and part of the triterpenoid constituents of *E. japonica* leaves, was collected and concentrated. To further enrich triterpenoid acids, the corresponding fraction was subjected to alkali treatment/appropriate purification based on differential solubility, and the resulting triterpenoid-acid-rich fraction was recovered after acidification and solvent removal. The flavonoid-rich fraction was obtained from the ethyl acetate fraction after concentration and drying. The obtained fractions were initially used in this study as operationally enriched triterpenoid-acid and flavonoid materials for subsequent co-amorphous preparation. However, because these materials were obtained by solvent partition and enrichment rather than isolation of chemically defined single compounds, they are more accurately described throughout this manuscript as a triterpenoid-acid-rich fraction and a flavonoid-rich fraction. No dedicated HPLC/UPLC compositional analysis was performed in the present study to verify whether ursolic acid and quercetin were the predominant constituents of these fractions. Therefore, these plant-derived materials should not be interpreted as chemically equivalent to the authentic ursolic acid–quercetin binary model system. In particular, because *E. japonica* leaves are known to contain corosolic acid and other structurally related triterpenoids, the triterpenoid-acid-rich fraction should be regarded as a multicomponent enriched fraction rather than an ursolic-acid-dominant material confirmed by direct analysis. Based on established mass ratios for preparing ursolic acid-quercetin amorphous complexes, co-amorphous complexes were synthesized from the purified *E. japonica* leaf components using physical grinding and solvent methods. For the physical grinding method, the triterpenoid-acid-rich fraction and flavonoid-rich fraction were mixed in mass ratios of 10:0.5, 10:1, 10:2, 10:5, and 10:10. A small volume of anhydrous ethanol was added, and the mixture was wet-ground for 2 h using a planetary ball mill to obtain the corresponding co-amorphous complex (referred to as the grinding-method complex). For the solvent method, the triterpenoid-acid-rich fraction and flavonoid-rich fraction were weighed in mass ratios of 10:0.2, 10:0.25, 10:0.5, 10:1, and 10:2. The triterpenoid-acid-rich fraction were dissolved in anhydrous ethanol using a water bath at 60 °C (DK-98-IIA, Tianjin Taist Instrument Co., Ltd., Tianjin, China), while the flavonoids were dissolved separately in anhydrous ethanol at 50 °C using the same water bath. The flavonoid solution was added to the triterpenoid acid solution under stirring for 10 min using a constant-temperature heating magnetic stirrer (DF-101S, Gongyi Yingyu Instrument Factory, Gongyi, China). Subsequently, the solvent was evaporated under reduced pressure with the rotary evaporator to yield the solid powder of the co-amorphous complex (referred to as the solvent-method complex). Accordingly, this fraction-based experiment was included only as an exploratory comparison and was not intended to provide the same level of mechanistic interpretation as the chemically defined ursolic acid–quercetin model system.

### 2.3. Determination of Ursolic Acid Content and Terpenoid Water Solubility

Using 5% vanillin-acetic acid and perchloric acid as chromogenic reagents, the content of ursolic acid was quantitatively determined by visible spectrophotometry (UV-2550, Shimadzu Corporation, Kyoto, Japan) at a wavelength of 545 nm, with ursolic acid standard as the reference. To evaluate the water solubility of the samples, accurately weighed ursolic acid standards and each prepared amorphous composite were dispersed in pure water. After dissolution with constant temperature water bath and ultrasonic assistance, they were reduced to supersaturation under reduced pressure, then filtered through a microporous membrane (0.45 μm, Millipore, Burlington, MA, USA). The filtrate was dried under water bath conditions. Finally, the content of ursolic acid in the authentic UA-containing samples, or the apparent total triterpenoid-acid equivalent content in the plant-derived samples, was determined using the aforementioned vanillin-acetic acid chromogenic method and calculated against the ursolic acid standard curve. Because this colorimetric assay is non-specific for individual triterpenoids, it does not distinguish ursolic acid from structurally related pentacyclic triterpenoid acids such as corosolic acid or oleanolic acid. Therefore, for the *E. japonica*-derived fraction-based samples, the resulting values should be interpreted as ursolic-acid-equivalent total triterpenoid content rather than a direct measurement of ursolic acid itself.

### 2.4. Structural Property Analysis of Amorphous Composites

The structural properties of the amorphous complexes were characterized by powder X-ray diffraction (PXRD) and Fourier-transform infrared (FTIR) spectroscopy. PXRD analysis was performed using a diffractometer (X’Pert, Malvern Panalytical, Almelo, The Netherlands) equipped with a Cu-Kα radiation source (λ = 1.5406 Å, 40 kV, 40 mA). The measurements were conducted in a 2θ range from 5° to 80° with a step size of 0.026° and a counting time of 36.465 s per step, corresponding to a scan rate of 4°/min. The patterns of ursolic acid monomer, quercetin monomer, the triterpenoid-acid-rich fraction, the flavonoid-rich fraction, and the corresponding co-processed systems were recorded. Only the formulations showing the best solubility performance were selected for PXRD and FTIR characterization, because these samples were considered the most representative candidates for assessing whether successful amorphization and intermolecular interactions had been achieved. FTIR spectra were obtained on a spectrometer (Nicolet iS10, Thermo Fisher Scientific, Waltham, MA, USA) using the KBr pellet method. Each sample was thoroughly mixed with dried KBr powder in an anhydrous environment and pressed into a transparent pellet. Spectra were acquired in the range of 4000–400 cm^−1^ with a resolution of 32 cm^−1^. The same set of samples as for PXRD was analyzed. DSC or other thermal analysis techniques were not included in this study; therefore, the thermal properties and storage stability of the obtained complexes were not directly evaluated. This limitation should be considered when interpreting the amorphous nature of the samples, which was inferred mainly from PXRD and supported by FTIR. In addition, because no chromatographic compositional characterization was performed for the *E. japonica*-derived fractions, the PXRD and FTIR results obtained for these plant-derived systems should be interpreted as bulk solid-state properties of enriched multicomponent fractions rather than structure-confirming evidence for a chemically defined ursolic acid–quercetin analog system.

### 2.5. Determination of the In Vivo Antitussive and Expectorant Activities of Ursolic Acid–Quercetin Co-Amorphous Systems

Seventy clean-grade male ICR mice (weight: 20 ± 2 g) were randomly divided into 7 groups (*n* = 10 per group): a blank control group, and experimental groups for ursolic acid monomer, the optimal co-grinding complex, and the optimal solvent-evaporation complex. Each experimental treatment was further subdivided into low-dose (2 mg/mL) and high-dose (4 mg/mL) groups. The antitussive and expectorant activities of the samples were evaluated using the ammonia-induced cough model and the tracheal phenol red secretion method, respectively. A quercetin-only group was not included in the present study. The study was designed to demonstrate the enhancement of ursolic acid’s therapeutic efficacy through co-amorphization with quercetin, leveraging quercetin’s ability to form interacting amorphous systems. Therefore, while quercetin may possess intrinsic pharmacological activity, the focus was on evaluating the overall efficacy of the co-amorphous formulations compared to ursolic acid alone. This limitation means that the specific contribution of quercetin to the observed pharmacological effects cannot be entirely distinguished from the effects of co-amorphization on ursolic acid delivery. The animal number used in this study was based on conventional group sizes adopted in exploratory in vivo pharmacological assays, and the present experiments were intended as an initial efficacy evaluation rather than a definitive confirmatory study. Ammonia-Induced cough test: Following a 10-day administration period (oral gavage, 0.05 mL/10 g body weight, once daily), the mice were placed individually under an inverted 500 mL beaker 1 h after the final dose. A cotton ball soaked with 0.2 mL of 25–28% (*v*/*v*) ammonia solution was quickly introduced into the beaker. The cough latency (time from ammonia exposure to the first cough) and the number of coughs within 3 min were recorded for each mouse. Tracheal phenol red secretion test: The dosing regimen was identical to the cough test. One hour after the final administration, mice were intraperitoneally injected with 0.5 mL of 0.5% (*w*/*v*) phenol red solution. After 30 min, the animals were euthanized. The trachea, from below the larynx to the bronchial bifurcation, was dissected and placed in a test tube containing 2 mL of normal saline. Then, 0.1 mL of 1 mol/L NaOH was added. The tube was kept in the dark for 30 min. The absorbance of the solution was measured at 546 nm using a UV-Vis spectrophotometer to quantify the phenol red concentration, which correlates with tracheal mucus secretion.

### 2.6. Data Analysis

For the analysis of differences in characteristic metabolite types, statistical analysis was conducted via SPSS software, version 26.0 (IBM Corp., Armonk, NY, USA). Data are presented as mean ± standard deviation (SD). For the pharmacological assays involving multiple groups, statistical comparisons were performed using one-way analysis of variance (ANOVA), followed by an appropriate post hoc multiple-comparison test. A value of *p* < 0.05 was considered statistically significant. Because this study was designed as an exploratory pharmacological investigation, no formal a priori power calculation was performed; therefore, the results should be interpreted as preliminary evidence that warrants further confirmation in larger, independently powered studies.

## 3. Results

### 3.1. Comparison of Water Solubility of Ursolic Acid-Quercetin Amorphous Complex

**Figure 1:**
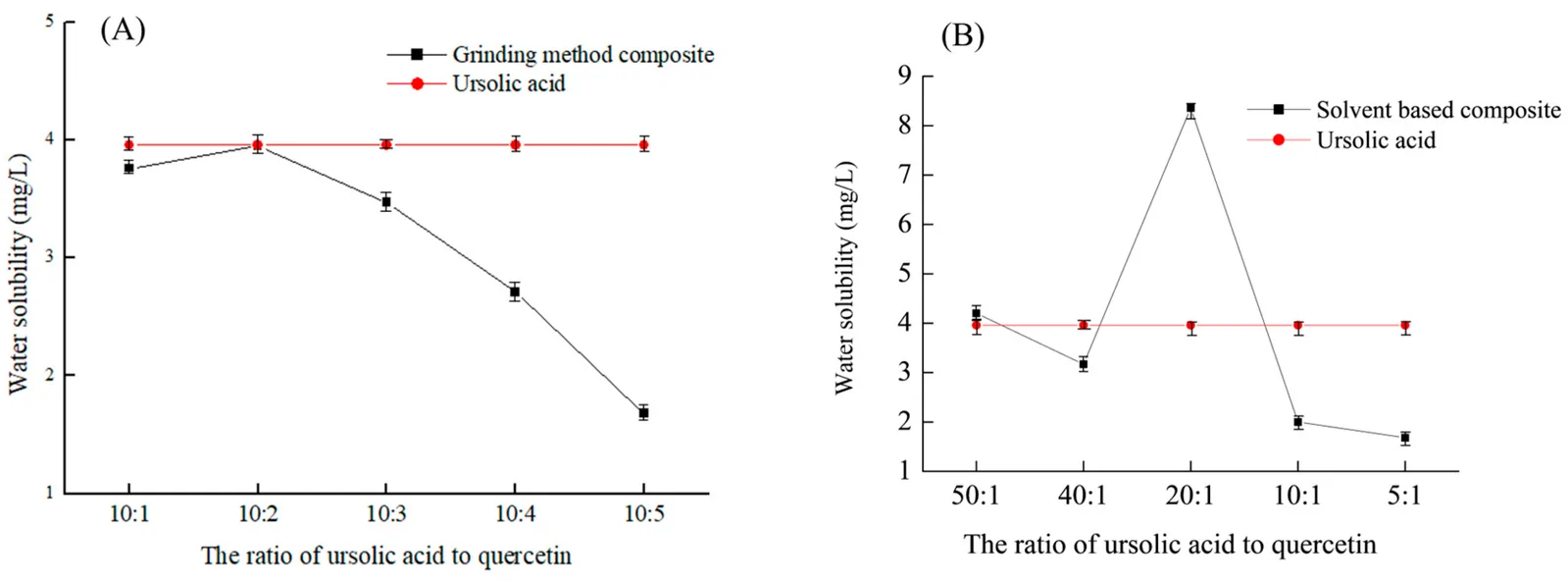
Comparison of the water solubility of ursolic acid and ursolic acid–quercetin systems prepared by (**A**) physical grinding and (**B**) solvent evaporation.

After dissolving the amorphous complex in water to obtain a saturated solution, the content of ursolic acid was determined using the vanillin-peracetic acid method. The results showed that the amorphous complex of quercetin and ursolic acid was prepared by the physical grinding method (Figure 1A). When the ratio of ursolic acid to quercetin was 5:1, the co-amorphous material prepared by physical grinding showed the highest water solubility. However, as the ratio of quercetin to ursolic acid increased, the water solubility decreased. This trend suggests that the solubility of the system is composition-dependent and reaches an optimum at a specific UA/QC ratio. In addition, it can also be seen from the figure that the water solubility of ursolic acid in the co-amorphous complex prepared by physical grinding was lower than that of monomeric ursolic acid, indicating that the effect of preparing by physical grinding was poor. The amorphous complex prepared by the solvent method (Figure 1B) achieved the best results when the ratio of ursolic acid to quercetin was 20:1. Both ursolic acid and quercetin are solid substances that are extremely difficult to dissolve in water. When the proportion of quercetin was too low, it may have been insufficient to promote effective co-amorphization with ursolic acid and thus failed to improve its water solubility. However, when the ratio of quercetin to ursolic acid increased, its water solubility decreased, which may be related to insufficient co-amorphization, excess quercetin remaining as a poorly soluble phase, or altered molecular packing in the solid dispersion; however, this mechanism was not directly investigated in the present study. At a mass ratio of ursolic acid to quercetin of 10:0.5, the solubility was markedly higher than that of ursolic acid alone, whereas at the other tested ratios, the solubility was lower than or comparable to that of the ursolic acid monomer.

### 3.2. Exploratory Solubility Evaluation of Co-Processed Plant-Derived Triterpenoid-Acid-Rich and Flavonoid-Rich Fractions

**Figure 2:**
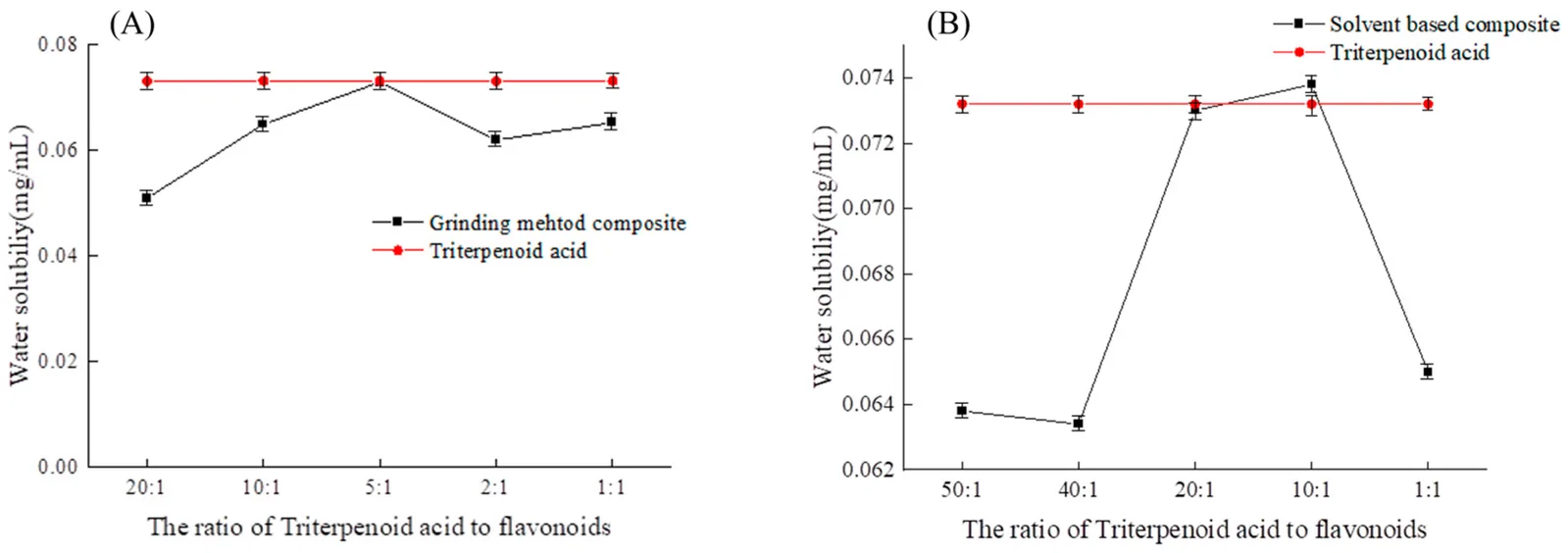
Comparison of the water solubility of co-processed systems prepared from the triterpenoid-acid-rich fraction and flavonoid-rich fraction of *E. japonica* leaves by (**A**) physical grinding and (**B**) solvent evaporation.

Amorphous complexes prepared by physical grinding, the amount of flavonoids affects the water solubility of the amorphous complexes (Figure 2A). When the ratio of triterpenoid acid to flavonoids is between 1:0.05 and 1:0.2, as the proportion of flavonoids increases, the water solubility of the amorphous complexes rises significantly, reaching a maximum of 0.073 mg/mL at a ratio of 1:0.2; when the ratio is between 1:0.2 and 1:0.5, the water solubility of the amorphous complexes decreases; and when the ratio is between 1:0.5 and 1:0.1, the water solubility of the amorphous complexes increases somewhat. Overall, when the ratio of triterpenoid acid to flavonoids is 1:0.2, the water solubility is best, comparable to the water solubility of triterpenoid acid without the addition of flavonoids. The amorphous complexes prepared by the solvent method also showed that the amount of flavonoids would affect the water solubility of the complexes (Figure 2B). Between the ratios of 1:0.05 to 1:0.5, an increase in the content of flavonoids improved the water solubility of triterpenoid acids in the complexes. However, further increasing the proportion of flavonoids reversed this effect and inhibited the water solubility of triterpenoid acids. At the optimal ratio of 1:0.5, the water solubility of the complexes reached a maximum value of 0.074 mg/mL, slightly exceeding the water solubility of triterpenoid acids without the addition of flavonoids. Although the improvement in water solubility was limited, this section remains important because it demonstrates that the effect of co-amorphization is strongly system-dependent and is influenced by the intrinsic composition and solid-state characteristics of the starting materials. In the case of *E. japonica* extracts, the limited benefit may be related to the partially amorphous nature of the purified fractions and the presence of residual impurities, which could reduce the extent of additional solubility gain after co-processing. Overall, co-processing the triterpenoid-acid-rich fraction with the flavonoid-rich fraction did not produce a clear or robust solubility enhancement under the present experimental conditions. Because the exact chemical compositions of both fractions were not established, these observations should be regarded only as preliminary formulation-level results. Therefore, in the revised manuscript, this fraction-based system is presented only as an exploratory comparison to the chemically defined UA–QC model system, rather than as an equivalent co-amorphous system for mechanistic interpretation.

### 3.3. Solid-State Characterization of the Ursolic Acid–Quercetin Co-Amorphous System

**Figure 3:**
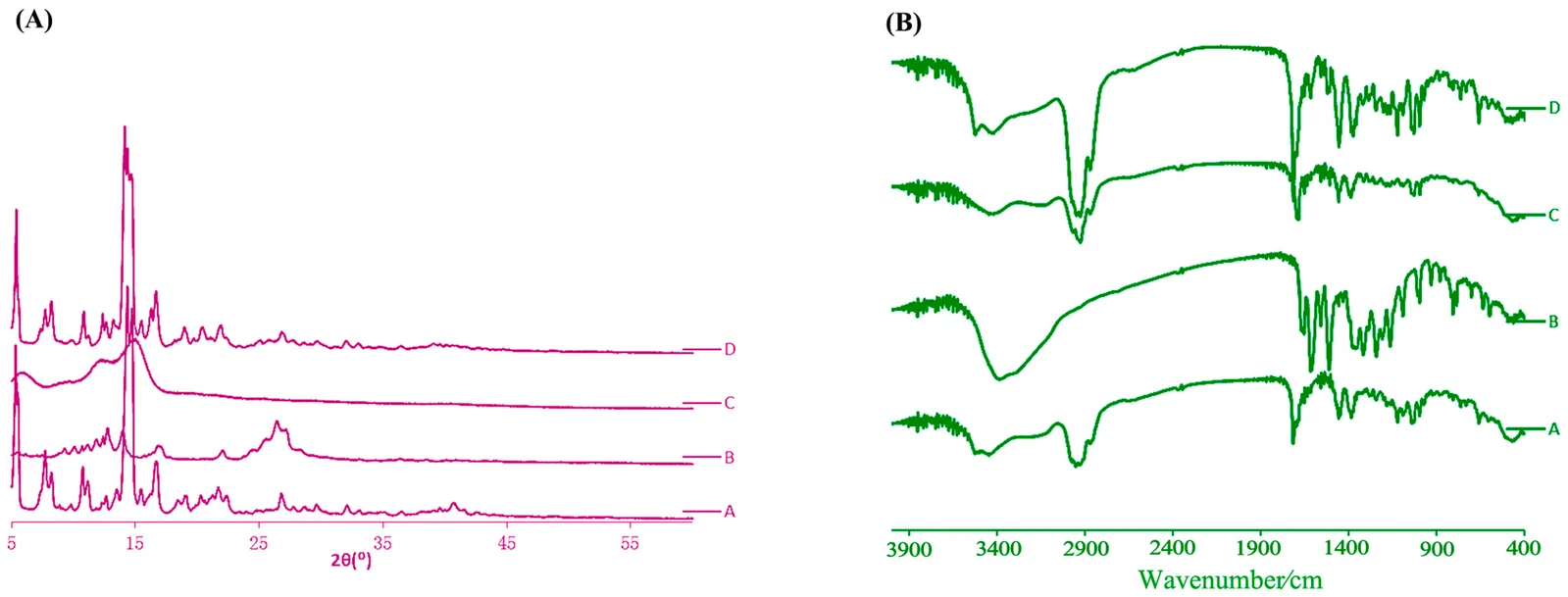
Solid-state characterization of the ursolic acid–quercetin system. (**A**) PXRD patterns of ursolic acid, quercetin, the ground mixture, and the solvent-prepared co-amorphous complex. (**B**) FTIR spectra of the corresponding samples. Note: A, ursolic acid; B, quercetin; C, physical mixture of ursolic acid and quercetin; D, co-amorphous ursolic acid–quercetin complex prepared by solvent evaporation.

The PXRD patterns of several compounds are presented in Figure 3A. Ursolic acid (UA) exhibited multiple sharp diffraction peaks within the 2θ range of 5.01° to 16.4°, characteristic of a crystalline material. Similarly, quercetin (QE) displayed strong crystalline peaks between 5.01° and 27.9°. The composite prepared by simple physical grinding (referred to as the ground mixture) also showed distinct crystalline peaks (5.01–25.3°), closely resembling those of the individual components. This indicates that physical mixing alone did not significantly alter the crystalline state of UA. In contrast, the pattern of the co-amorphous composite prepared via the solvent evaporation method showed notably broadened and attenuated diffraction peaks. Several characteristic peaks of both UA and QE disappeared, suggesting the formation of a predominantly amorphous state for both components within this complex. However, because DSC was not performed, the glass transition behavior and thermal stability of the amorphous phase could not be confirmed experimentally in the present study.

FTIR spectra of the samples are shown in Figure 3B. The spectrum of pure UA featured characteristic bands at 3448 cm^−1^ (assigned to free O-H stretching vibrations), 1716 cm^−1^ (C=O stretching), 1701 cm^−1^ (C=C stretching), and a strong signal at 2952 cm^−1^ (C-H stretching of CH_2_ groups). For pure QE, key bands were observed at 3385 cm^−1^ (broad band for O-H stretching), 1654 cm^−1^ (C=O stretching), and at 1617 cm^−1^ and 1512 cm^−1^ (aromatic C=C ring stretching). Significant spectral changes were observed in the UA-QE co-amorphous complex (solvent method). The broad O-H stretching band shifted slightly to 3447 cm^−1^, which is hypothesized to result from the involvement of hydroxyl groups as hydrogen bond donors. The carbonyl band of UA shifted minimally to 1717 cm^−1^, while the C=O band of QE remained at 1654 cm^−1^, albeit with weaker intensity due to its lower relative proportion in the complex. These small shifts and intensity changes suggest altered intermolecular environments, but they should be interpreted as indirect evidence of molecular interactions rather than definitive proof of hydrogen-bond formation. The intensities of the aromatic ring signals (QE) were also reduced. Taken together with the PXRD result showing marked attenuation and disappearance of crystalline peaks, the FTIR data support the formation of a predominantly amorphous state accompanied by probable intermolecular interactions between UA and QE. The FTIR spectrum of the ground mixture shared general similarities with that of the co-amorphous complex, except in the hydroxyl region. Here, the O-H bands of UA and QE were observed at 3524 cm^−1^ and 3421 cm^−1^, respectively, showing a clear separation and an upfield shift compared to their pure states (3448 cm^−1^ for UA and 3385 cm^−1^ for QE). However, because PXRD indicated that the ground mixture remained largely crystalline, these FTIR shifts alone are insufficient to confirm co-amorphization or strong intermolecular bonding. The lack of a merged, shifted O-H band in the ground mixture suggests the absence of extensive intermolecular hydrogen bonding between UA and QE, consistent with the two components remaining largely as separate crystalline entities, as evidenced by PXRD.

### 3.4. Exploratory Solid-State Analysis of Co-Processed Plant-Derived Triterpenoid-Acid-Rich and Flavonoid-Rich Fractions

**Figure 4:**
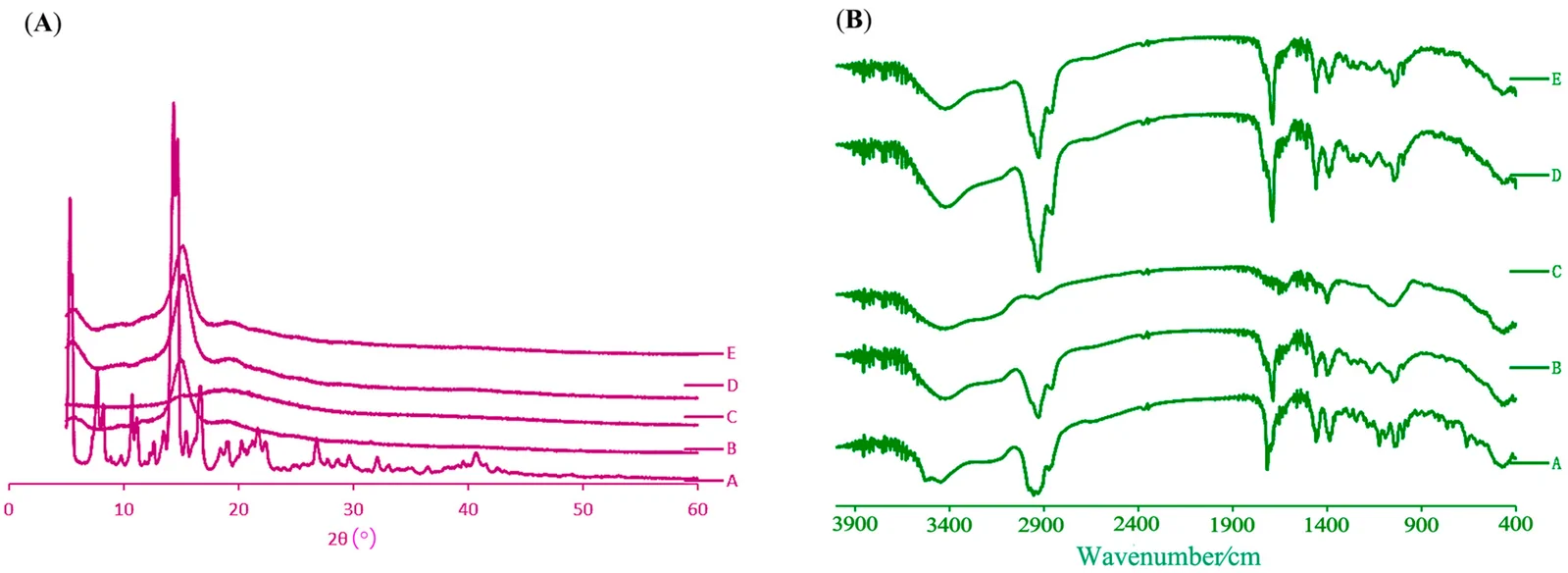
Structural and performance analysis of amorphous complexes of triterpenoid acids and flavonoids in *E. japonica* leaves; (**A**) _PXRD pattern of triterpenoid acid-flavonoid in *E. japonica* leaf; (**B**) _FTIR spectrum of the amorphous complex of triterpenoid acid-flavonoid in *E. japonica* leaf. Note: A—ursolic Acid; B—triterpenoid acid; C—flavonoid; D—triterpenoid acid-flavonoid physical mixture (20:1); E- triterpenoid acid-flavonoid amorphous complex (20:1).

The Powder X-ray Diffraction (PXRD) patterns of various samples are presented in Figure 4A. Ursolic acid (UA) displayed characteristic diffraction peaks within the 2θ range of 11.9° to 16.4°, confirming its crystalline nature. In contrast, the dominant diffraction peaks associated with triterpenoid acids (TAs) were absent, suggesting a largely amorphous or poorly crystalline state rather than allowing a definitive distinction between a truly amorphous phase and low crystallinity caused by multicomponent interference or impurity broadening. PXRD patterns of flavonoids (FL), physical mixtures of TAs-FL (20:1 ratio), and co-amorphous mixtures of TAs-FL (20:1 ratio, prepared by grinding) all indicated an amorphous state for the FL component. However, because PXRD is insensitive to trace crystalline domains and cannot reliably quantify amorphous content in complex mixtures, these data should be interpreted as evidence of reduced long-range order rather than proof of a single-phase amorphous material. In the absence of DSC, solid-state NMR, or quantitative crystalline phase analysis, the possibility that the observed pattern arose from low crystallinity, phase dilution, or overlapping impurity signals cannot be excluded. Accordingly, the possibility that residual flavonoids or other co-extracted components remained in the TA fraction should be regarded as an inference based on the structural data rather than as a directly measured result. At present, the study does not include dedicated chromatographic compositional profiling (e.g., HPLC/UPLC) to define the exact chemical composition of either the triterpenoid-acid-rich fraction or the flavonoid-rich fraction. Therefore, the PXRD patterns of these plant-derived samples cannot be interpreted at the level of individual major compounds with confidence. In particular, it was not possible to verify whether ursolic acid was the dominant triterpenoid, whether corosolic acid was present in substantial proportion, or whether additional flavonoids and phenolic constituents contributed materially to the broad diffraction background. Accordingly, these structural data should be regarded as describing the bulk solid-state features of enriched multicomponent fractions rather than those of a chemically defined two-component co-amorphous system. Therefore, the hypothesis that TA-FL interactions may have pre-existed in the crude extract remains tentative and requires further validation.

Fourier Transform Infrared (FTIR) spectra of the samples are shown in Figure 4B. Using UA as a reference spectrum, the FTIR spectrum of the TA fraction from loquat leaves was analyzed. Characteristic functional groups were identified: a broad signal at 3421 cm^−1^ (likely attributable to adsorbed water, with minimal contribution from free O-H groups), an olefinic group at 1685 cm^−1^ (C=C stretching), a carboxyl group (COOH) at 1750 cm^−1^ (overlapping with a carbonyl group), and three carbonyl groups at 1713 cm^−1^ (C=O stretching). For FL, characteristic bands were observed at 3447 cm^−1^ (strong, attributed to O-H and phenolic O-H stretching), 1685 cm^−1^ (C=O stretching), and at 1507 cm^−1^ and 1617 cm^−1^ (aromatic C=C ring stretching). The FTIR spectrum of the co-amorphous TA-FL complex (20:1 ratio) prepared by mechanical grinding exhibited an O-H stretching band at 3421 cm^−1^, identical to that of the TA fraction. This observation might be due to the large compositional ratio difference between TA and FL, where the FL’s O-H signal is obscured or overlapped by the more abundant TA’s signal. The carbonyl stretching vibrations, originally observed at 1713 cm^−1^ (TA) and 1685 cm^−1^ (FL), showed slight hypsochromic shifts to 1717 cm^−1^ (TA-derived) and 1690 cm^−1^ (FL-derived), respectively. Crucially, the characteristic peaks of the benzene rings at 1507 cm^−1^ and 1617 cm^−1^ remained unshifted. This lack of spectral shift in the aromatic region suggests that no significant intermolecular bonding or complexation occurred between the two compounds in the ground mixture. In contrast, the FTIR spectrum of the co-amorphous TA-FL complex (20:1 ratio) prepared by the solvent method displayed a significant shift in the original carbonyl signals at 1713 cm^−1^ and 1685 cm^−1^ to a sharper peak at 1717 cm^−1^. This unified and shifted carbonyl signal indicates that the carbonyl groups from both TA and FL participated in the formation of the complex structure. The observed hypsochromic shifts in these carbonyl signals further support the hypothesis of dimer formation or intermolecular interaction between the two components. Nevertheless, because the residual impurity content was not quantitatively determined, these spectral interpretations should be viewed as supportive but not definitive evidence for pre-existing amorphous associations in the extract.

### 3.5. In Vivo Antitussive and Expectorant Activities of the Ursolic Acid–Quercetin Systems

In the preliminary experiment, the experimental groups were administered ursolic acid at a concentration of 1 mg/mL, as well as composite preparations prepared via grinding and solvent methods, administered by gavage. All three treatment groups significantly extended the cough latency period compared to controls but did not produce a significant reduction in cough frequency or phenol red secretion. When the concentration of the composite preparations was increased to 2 mg/mL, the treatments markedly enhanced phenol red secretion in the mouse respiratory tract, indicating that higher doses of ursolic acid-quercetin amorphous composites exerted notable antitussive effects. Based on these findings, doses of 2 mg/mL (low-dose group) and 4 mg/mL (high-dose group) were selected for the subsequent formal experiments. The results are summarized in Table 1. Except for the high-dose ursolic acid monomer group, which showed no significant difference from the blank control group, all other treatment groups significantly prolonged the cough latency period. Regarding cough frequency, the high-dose solvent method composite displayed a significant reduction compared to the blank control. Both the grinding method composite and the high-dose solvent method composite demonstrated a significant increase in phenol red secretion relative to the blank control and the ursolic acid monomer group (*p* < 0.05). Although the solvent-prepared complex exhibited a more pronounced improvement in water solubility than the grinding-prepared complex, both formulations showed superior in vivo antitussive and expectorant activities compared with ursolic acid alone. This suggests that the biological efficacy of the co-amorphous systems may not depend exclusively on equilibrium aqueous solubility, but may also be influenced by improved wetting, altered solid-state properties, enhanced dispersion, and transient supersaturation during dissolution. However, because no in vitro dissolution study under biorelevant conditions, permeability assay, or in vivo pharmacokinetic evaluation was performed, the present data do not demonstrate that the observed pharmacological improvement resulted from enhanced oral bioavailability. Accordingly, the current results should be interpreted as showing an association between formulation-induced physicochemical changes and improved in vivo activity, rather than as direct evidence of a bioavailability-mediated mechanism. Therefore, the present results support the conclusion that both complexes can enhance pharmacological activity, while the solvent-prepared system appears to be more favorable for solubility improvement. In summary, the antitussive and expectorant effects of the composites prepared via grinding and solvent methods were comparable and both were superior to those of ursolic acid monomer alone. However, the degree of solubility enhancement was not identical between the two systems, and this should be considered when interpreting the relationship between physicochemical properties and biological activity.

## 4. Discussion

To address the challenges associated with poorly water-soluble drugs, co-amorphous systems have emerged as a relatively novel strategy [18]. Traditional drug-polymer amorphous solid dispersions improve solubility but are often limited by high polymer loadings, which result in increased dosage size and can introduce other formulation challenges like poor compressibility and flowability [19]. In contrast, drug–drug co-amorphous systems, typically composed of two active pharmaceutical ingredients (APIs), offer a promising alternative. These systems can be prepared via co-grinding, quench cooling, or other methods and are stabilized by potential intermolecular interactions between the two APIs [20]. While quench cooling may induce thermal degradation, and ball milling or cryo-milling can be time-consuming [21], solvent methods offer a simpler, more rapid alternative. Solvent evaporation often facilitates more complete amorphization by allowing rapid formation of stabilized molecular co-dispersions [22].

The central objective of the present study was to evaluate the ursolic acid–quercetin (UA–QC) system as a chemically defined co-amorphous model for improving the formulation performance of a poorly soluble triterpenoid acid. Within this framework, the UA–QC system provided the clearest basis for interpretation because both components were authentic reference compounds with known identities and controlled composition. Powder X-ray diffraction (PXRD) analysis indicated that the solvent method promoted the formation of a co-amorphous composite, whereas physical grinding was less effective in achieving a fully amorphous co-dispersed state [23]. Fourier-transform infrared (FTIR) spectroscopy further supported these findings: ground composites presented separate hydroxy group signals for UA and QC, without evidence of hydrogen bonding, indicating largely separate molecular entities [24]. In contrast, the solvent-processed composite showed spectroscopic shifts consistent with hydrogen bond formation between UA and QC, confirming interaction between the two compounds, which is in agreement with PXRD observations [25]. However, we emphasize that these FTIR shifts are modest and should be regarded as supportive evidence of altered molecular interactions rather than conclusive proof of a unique hydrogen-bonding network. The designation of the solvent-processed sample as a co-amorphous system is primarily supported by the loss of crystalline diffraction peaks in PXRD, together with the FTIR-indicated changes in functional-group environments. We acknowledge that DSC was not performed in this study; therefore, the thermal characteristics, including the glass transition temperature and physical stability of the co-amorphous system, remain to be verified in future work. Importantly, the biological results do not correlate strictly with equilibrium water solubility. Although the solvent-prepared formulation exhibited the clearest solubility improvement, both co-amorphous systems enhanced antitussive and expectorant activity relative to ursolic acid alone. This indicates that in vivo performance may also be influenced by formulation-dependent factors such as reduced crystallinity, improved wettability, enhanced dispersion, and transient supersaturation during dissolution. While quercetin itself possesses known pharmacological activities, the absence of a quercetin-only control group in our study means we cannot definitively quantify quercetin’s specific contribution to the observed effects. However, the significant improvements seen with both UA-QC formulations compared to pure ursolic acid, particularly in conjunction with the enhanced physicochemical properties (amorphization, improved solubility) of the co-amorphous systems, strongly suggest that co-amorphization plays a crucial role in facilitating the delivery and thus enhancing the in vivo efficacy of ursolic acid. Therefore, the present pharmacodynamic data support an overall improvement in biological efficacy for both formulations as co-amorphous systems designed to improve ursolic acid delivery, rather than definitively proving distinct pharmacological contributions from each component. Moreover, because no dissolution testing in biorelevant media, membrane permeability evaluation, or plasma pharmacokinetic analysis was conducted, the present study cannot directly confirm that the improved pharmacological activity resulted from enhanced oral bioavailability. The current findings should therefore be interpreted as an association between formulation modification, solubility-related properties, and in vivo efficacy, rather than as definitive mechanistic proof of the individual components’ pharmacological roles. Likewise, the lack of a clear dose–response relationship in the ursolic acid monomer group suggests that nominal dose escalation alone may be insufficient to improve in vivo efficacy for a poorly soluble compound. This phenomenon may be related to dissolution-limited absorption, possible saturation of effective exposure within the tested dose range, or experimental variability in the animal model. Accordingly, broader dose-ranging studies combined with dissolution and pharmacokinetic evaluation will be necessary to determine whether the observed nonlinearity reflects limited gastrointestinal availability or other biological factors. Taken together, the current evidence suggests that the solvent method was more favorable than grinding for promoting amorphization and improving solubility-related properties in the UA-QC system under the present preparation conditions. However, the in vivo antitussive and expectorant data showed only limited differences between the two formulations. Therefore, our results do not justify a broad or universal conclusion that the solvent method is categorically superior to the grinding method in overall pharmaceutical performance. Rather, the available evidence supports a more cautious interpretation: the solvent method appears advantageous primarily at the physicochemical level, whereas both preparations produced broadly comparable biological benefits in the tested animal model. Accordingly, any conclusion regarding the superiority of one preparation method over the other should be considered formulation-specific and endpoint-dependent, pending confirmation by additional comparative studies involving dissolution behavior, physical stability, and pharmacokinetic performance. These observations are consistent with the possibility that formulation-induced changes in dissolution-related behavior contributed to the improved in vivo performance; however, this mechanism remains to be verified experimentally. Nevertheless, the findings support the potential utility of co-amorphous systems for improving the formulation performance of poorly soluble compounds such as UA [26].

Separately, experiments were conducted to explore the formation of co-amorphous composites between triterpenoid acids (TAs) and flavonoids (FLs) purified from loquat leaves using similar grinding and solvent methods. Notably, the resulting composites did not significantly enhance the water solubility of TAs [27]. PXRD and FTIR analysis revealed that both the purified TAs and FLs existed in an amorphous state and contained substantial residual impurities [28]. This suggests that TA-FL interactions may pre-exist in the crude extract, forming an amorphous complex during extraction [29]. Consequently, additional attempts to form a co-amorphous composite did not yield further solubility benefits. Nevertheless, as with the UA-QC system, the solvent method appeared to facilitate intermolecular interactions more effectively than grinding at the solid-state characterization level, but this observation should not be overextended to imply universal superiority across all functional endpoints [30]. These results highlight the importance of starting material purity and pre-existing molecular associations when designing co-amorphous formulations. For compounds already present in an amorphous complex in crude extracts, traditional co-processing may not provide additional solubility advantages [31]. Thus, in cases where discrete components can be effectively isolated and combined, the solvent method may represent a useful route for promoting co-amorphization and solubility enhancement, although its practical superiority over physical grinding should be judged on a case-by-case basis [32]. It should be noted that, when moving from the chemically defined UA–QC model system to the plant-derived triterpenoid-acid-rich fraction/flavonoid-rich fraction system of *E. japonica*, two variables changed simultaneously. Therefore, the present design does not allow the individual effects of triterpenoid-fraction composition and flavonoid-fraction composition on amorphization, intermolecular interactions, or solubility to be distinguished clearly. Future studies should include the cross-combination systems of ursolic acid–flavonoid-rich fraction and triterpenoid-acid-rich fraction–quercetin to better clarify the role of fraction composition. Because these experiments were not performed in the present study, this should be considered a limitation and a direction for future research. The present study has several limitations. The enriched fractions derived from *E. japonica* were not chemically characterized by HPLC/UPLC or LC-MS, and no plasma pharmacokinetic evaluation, in vitro dissolution testing under biorelevant conditions, or particle-size/wettability analysis of the final dosage forms was performed. Therefore, this plant-derived system should be regarded as an exploratory multicomponent formulation model rather than a confirmed ursolic acid–quercetin equivalent system, and the contribution of factors other than equilibrium solubility to the enhanced antitussive and expectorant effects cannot be fully excluded. Future studies should integrate qualitative and quantitative analysis of the major constituents, supersaturation/dissolution profiling, permeability testing, and pharmacokinetic evaluation to more rigorously elucidate the relationships among composition, amorphization behavior, solubility enhancement, and pharmacodynamic improvement. In particular, future experimental designs should include the cross-combination systems of ursolic acid–flavonoid-rich fraction and triterpenoid-acid-rich fraction–quercetin, so that the respective contributions of triterpenoid-fraction composition and flavonoid-fraction composition can be distinguished more clearly.

## 5. Conclusions

This study demonstrates that co-amorphous complexation of ursolic acid and quercetin is a feasible strategy for improving the formulation performance of a poorly soluble triterpenoid acid. Among the two preparation methods examined, solvent evaporation produced a UA–QC system with more evident reduction in crystallinity, stronger indication of intermolecular interaction, and higher water solubility than physical grinding. Both UA–QC formulations also enhanced in vivo expectorant and antitussive activity compared with ursolic acid alone, although the underlying bioavailability mechanism was not directly established. In contrast, the plant-derived triterpenoid-acid-rich fraction/flavonoid-rich fraction system showed only limited solubility improvement and should be regarded as a preliminary exploratory comparison because its chemical composition was not fully characterized. Therefore, the principal conclusion of the present work is centered on the chemically defined UA–QC model system. Further studies on dissolution behavior, physical stability, pharmacokinetics, and compositionally resolved plant-derived systems are needed.

## Figures and Tables

**Table 1:** Antitussive and Expectorant Activities of Ursolic Acid and Its Preparations (n = 10 per group).

Sample	Concentration (mg/mL)	Cough Incubation Period (s)	Number of Coughs (3 min)	Phenol Red Concentration (mg/mL)
Control	-	5.43 ± 2.44 a	26.00 ± 6.20 bc	0.054 ± 0.019 a
Ursolic acid	2	17.67 ± 4.55 b	30.29 ± 6.24 c	0.052 ± 0.017 a
	4	12.14 ± 7.38 ab	25.00 ± 5.40 bc	0.052 ± 0.023 a
Milling Method Complex	2	19.78 ± 7.03 b	23.00 ± 6.87 ab	0.083 ± 0.017 bc
	4	20.00 ± 9. 34 b	20.88 ± 4.36 ab	0.097 ± 0.027 c
Solvent-based complex	2	13.88 ± 5.87 b	22.11 ± 5.62 ab	0.066 ± 0.017 ab
	4	20.38 ± 9.68 b	18.29 ± 5.31 a	0.082 ± 0.029 bc

Note: Values are expressed as mean ± SD. Different letters within the same column indicate significant differences among groups (*p* < 0.05).

## Data Availability

The original contributions presented in this study are included in the article. Further inquiries can be directed to the corresponding authors.
